# Supplementary figures and images for: Developing a clinically relevant radiosensitizer for temozolomide-resistant gliomas
Source: PLoS One. 2020 Sep 3;15(9):e0238238. doi: 10.1371/journal.pone.0238238 (PMC7470340; doi:10.1371/journal.pone.0238238)

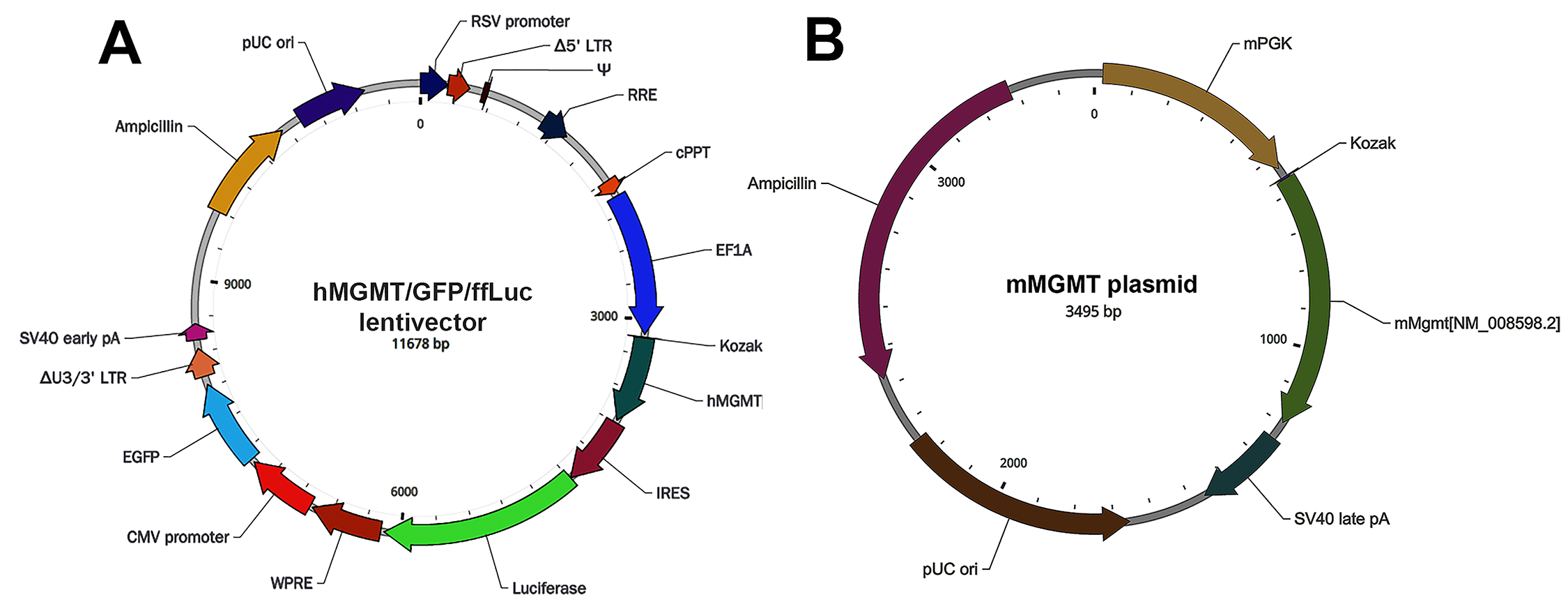

Supplement: S1 Fig — The LN229M and U251M cell variants were generated after infection with a lentiviral construct that carries the CDS for human MGMT in tandem with the one for firefly luciferase separated by an IRES sequence and under an EF1α promoter and, in a separate ORF on the same lentivector, the CDS for enhanced GFP under a CMV promoter (Panel A). The GFP reporter facilitated the selection of high MGMT expressing variants of these GB cells which were sorted to purity by FACS. The GL261M cell variant was generated after transfection with a plasmid construct that carries the murine Mgmt CDS under the control of a murine phosphoglycerate kinase (mPGK) promoter (Panel B). The transfected cells were further enriched in murine Mgmt by serial passaging in medium containing increasing concentrations of TMZ. (TIF) [file pone.0238238.s001.tif]

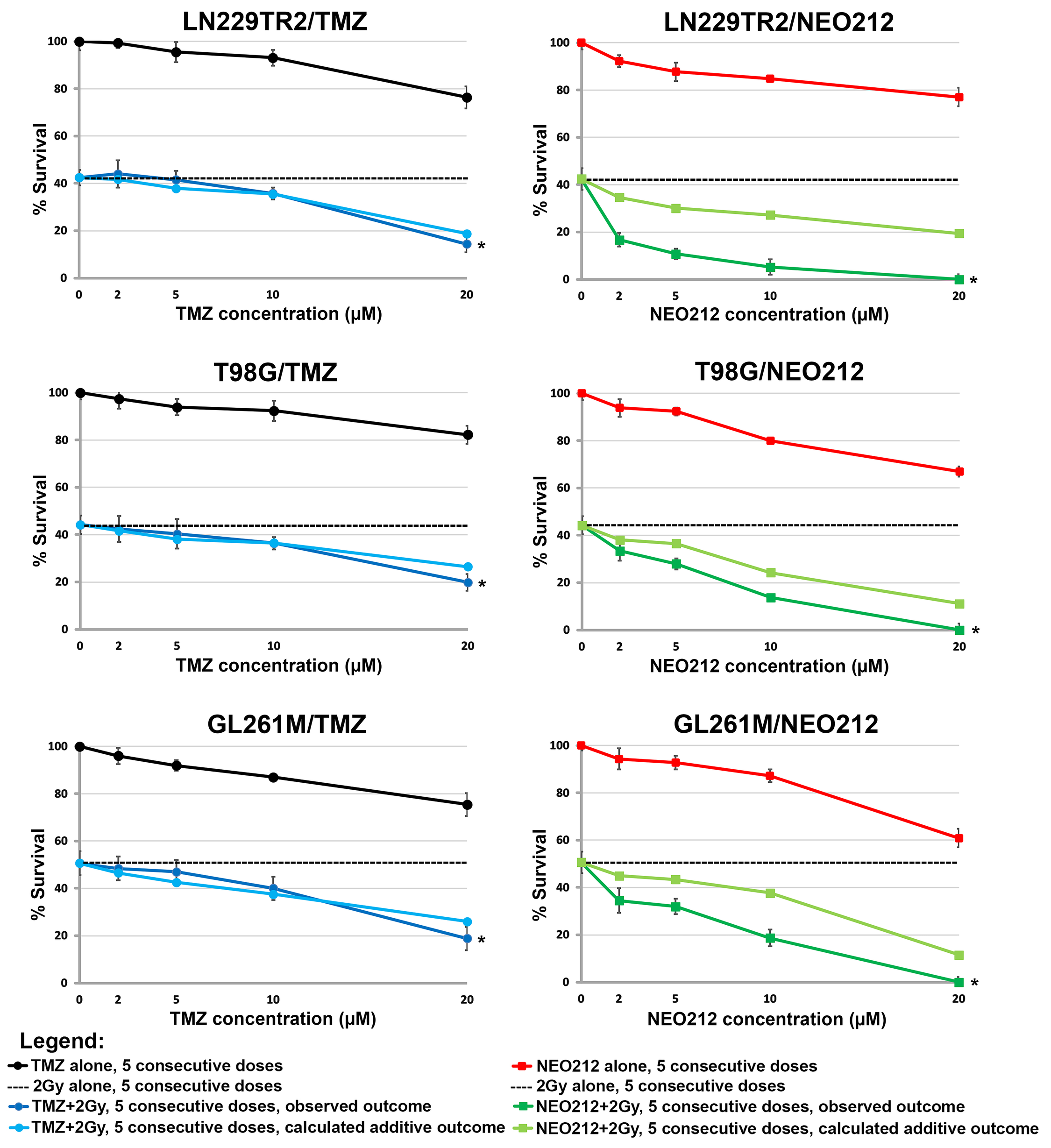

Supplement: S2 Fig — In these CFA analyses, we tested both drugs using a treatment schedule designed to mimic the Stupp protocol (i.e., five consecutive days of concurrent chemotherapy plus radiotherapy vs. five consecutive days of monotherapies). The colony survival data show that NEO212 can synergize with ionizing radiation in the clinically relevant concentration range (i.e., 10 μM or less), whereas TMZ becomes synergistic only outside (i.e., >10 μΜ) of this concentration range where presumably achieves levels of DNA alkylation optimal for synergistic effects to take place (* indicates a p<0.01 determined by Student’s t-test). (TIF) [file pone.0238238.s002.tif]

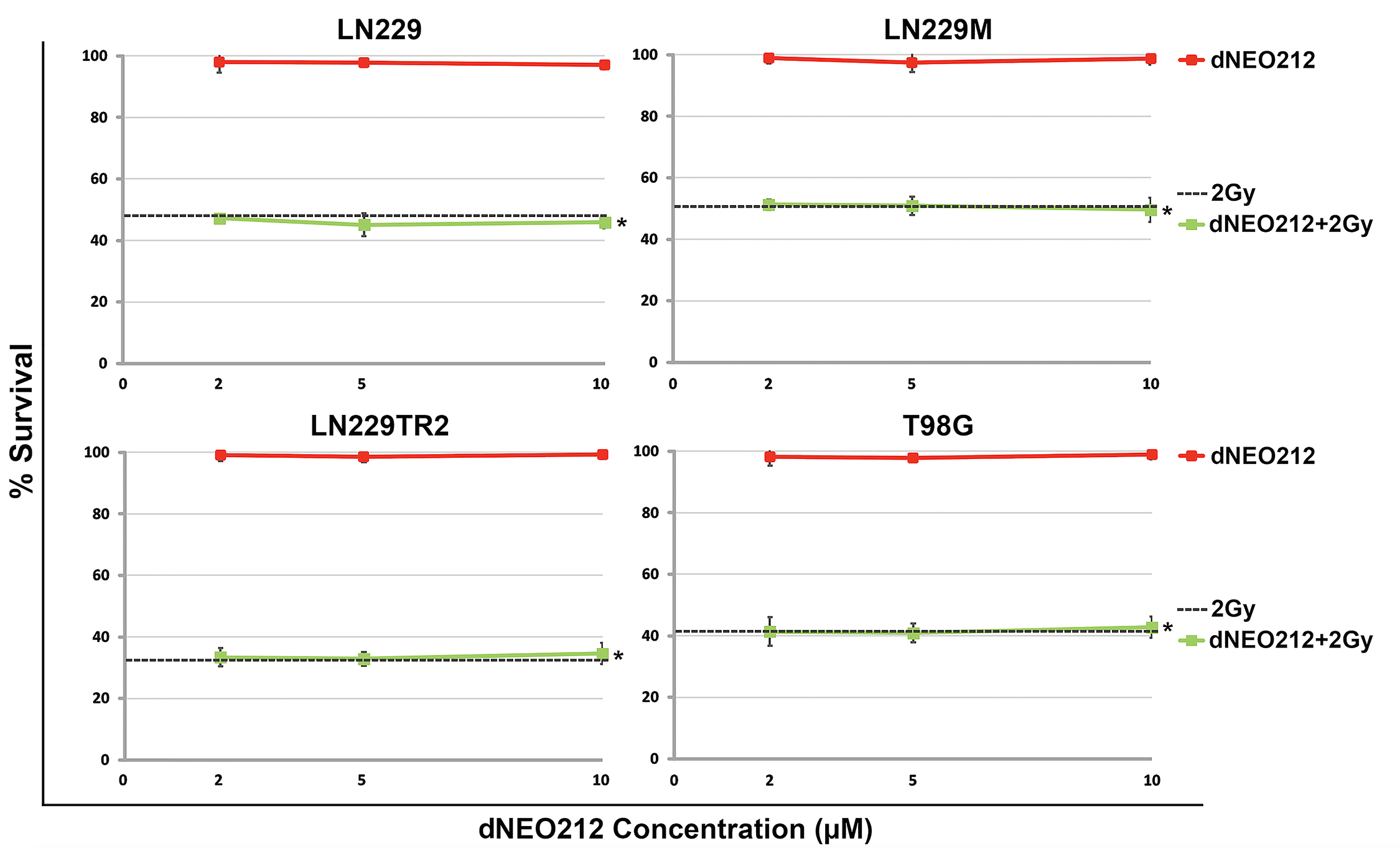

Supplement: S3 Fig — If NEO212 is pre-incubated in medium for 24hrs (i.e., by becoming decayed NEO212 or dNEO212) before is added to the cells, it loses all of its cytotoxic and radiosensitization properties. In these CFA analyses, we tested dNEO212 using the same treatment schedule designed to mimic the Stupp protocol (i.e., five consecutive days of treatments). The colony survival data show that dNEO212 completely lost its cytotoxicity even against TMZ-sensitive cells (* indicates a p<0.01 determined by Student’s t-test). (TIF) [file pone.0238238.s003.tif]

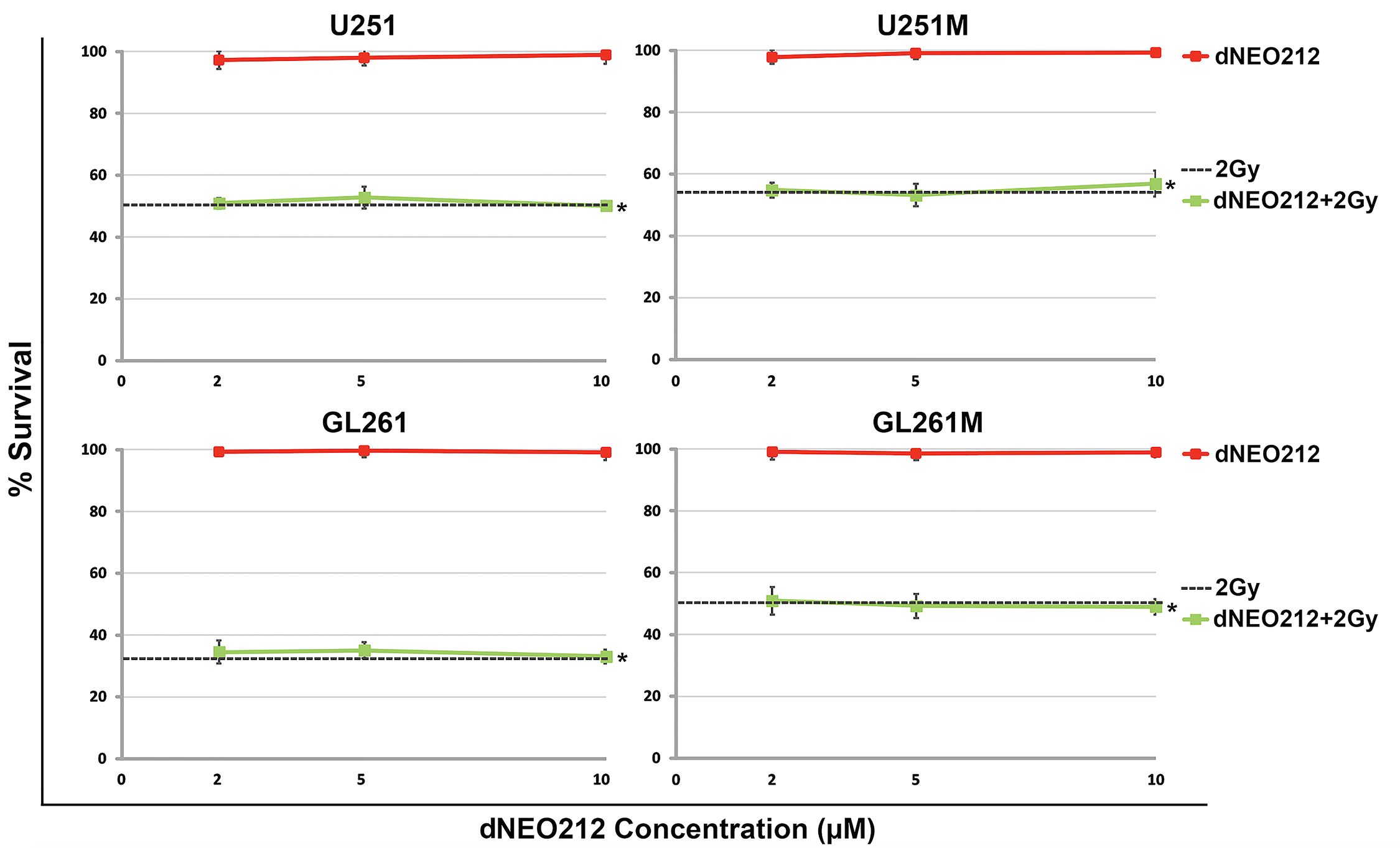

Supplement: S4 Fig — If NEO212 is pre-incubated in medium for 24hrs (i.e., by becoming decayed NEO212 or dNEO212) before is added to the cells, it loses all of its cytotoxic and radiosensitization properties. In these CFA analyses, we tested dNEO212 using the same treatment schedule designed to mimic the Stupp protocol (i.e., five consecutive days of treatments). The colony survival data show that dNEO212 completely lost its cytotoxicity even against TMZ-sensitive cells (* indicates a p<0.01 determined by Student’s t-test). (TIF) [file pone.0238238.s004.tif]

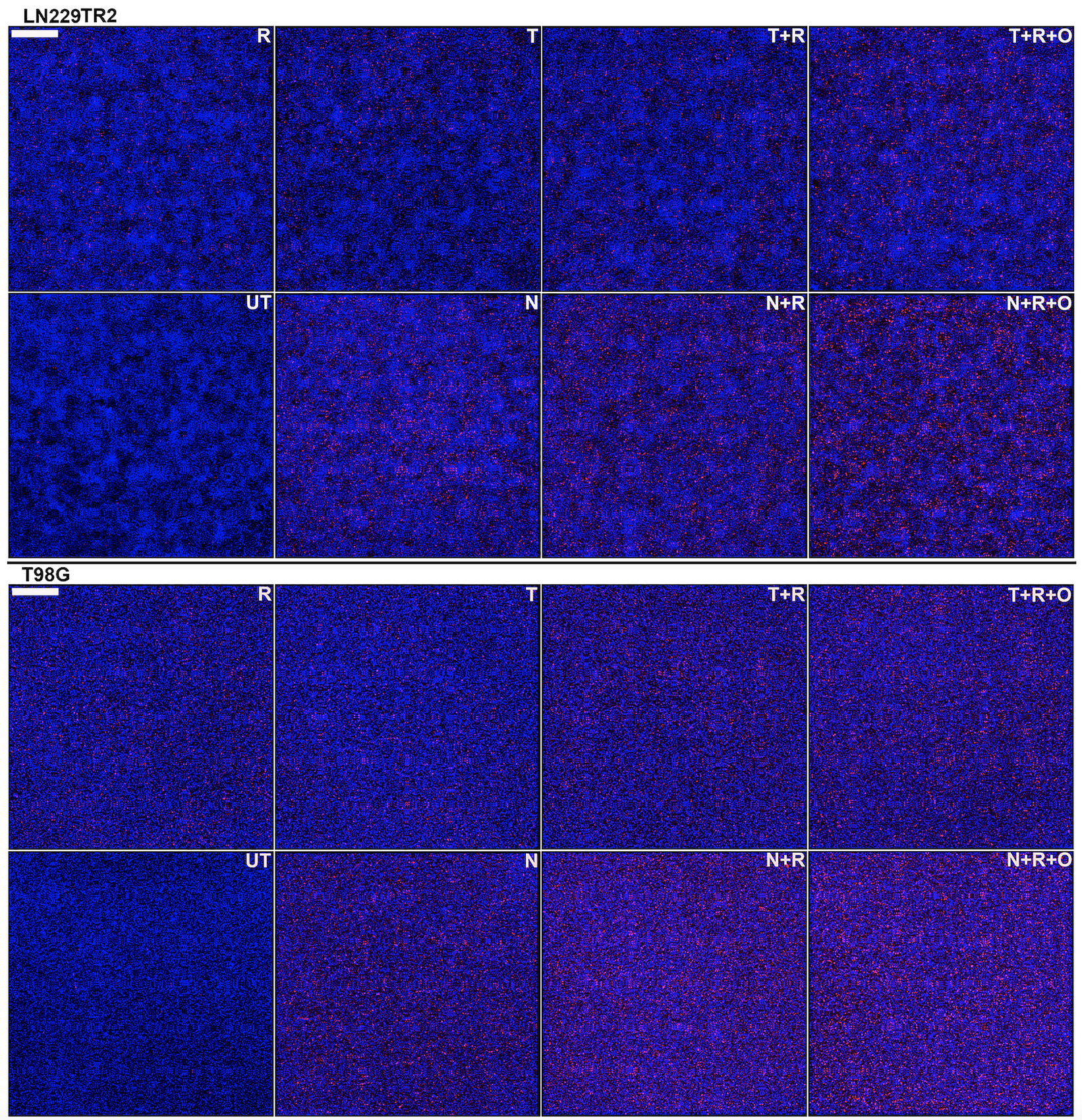

Supplement: S5 Fig — LN229TR2 (a MMR-deficient variant of LN229 cells) and T98G (an endogenously expressing MGMT line) cells were seeded at high densities (50,000 cells/cm2) and either left untreated (UT) or treated for five consecutive days with either 10 μM TMZ (T) or NEO212 (N) or 2 Gy (R) alone or combinations without (T+R or N+R) or with (T+R+O or N+R+O) Olaparib (O). The cells were probed with a γH2AX antibody and an AF647-labeled secondary and nuclei were counterstained with DAPI. Persistent γH2AX foci (red) were digitally counted relative to the total number of cell nuclei (blue). Each panel is data dense and represents a composite of 36 fields in total (i.e., a square of about 3x3 mm) captured on a widefield microscopy instrument and digitally stitched together. Scale bar is 500 μm (upper left corner). (TIF) [file pone.0238238.s005.tif]

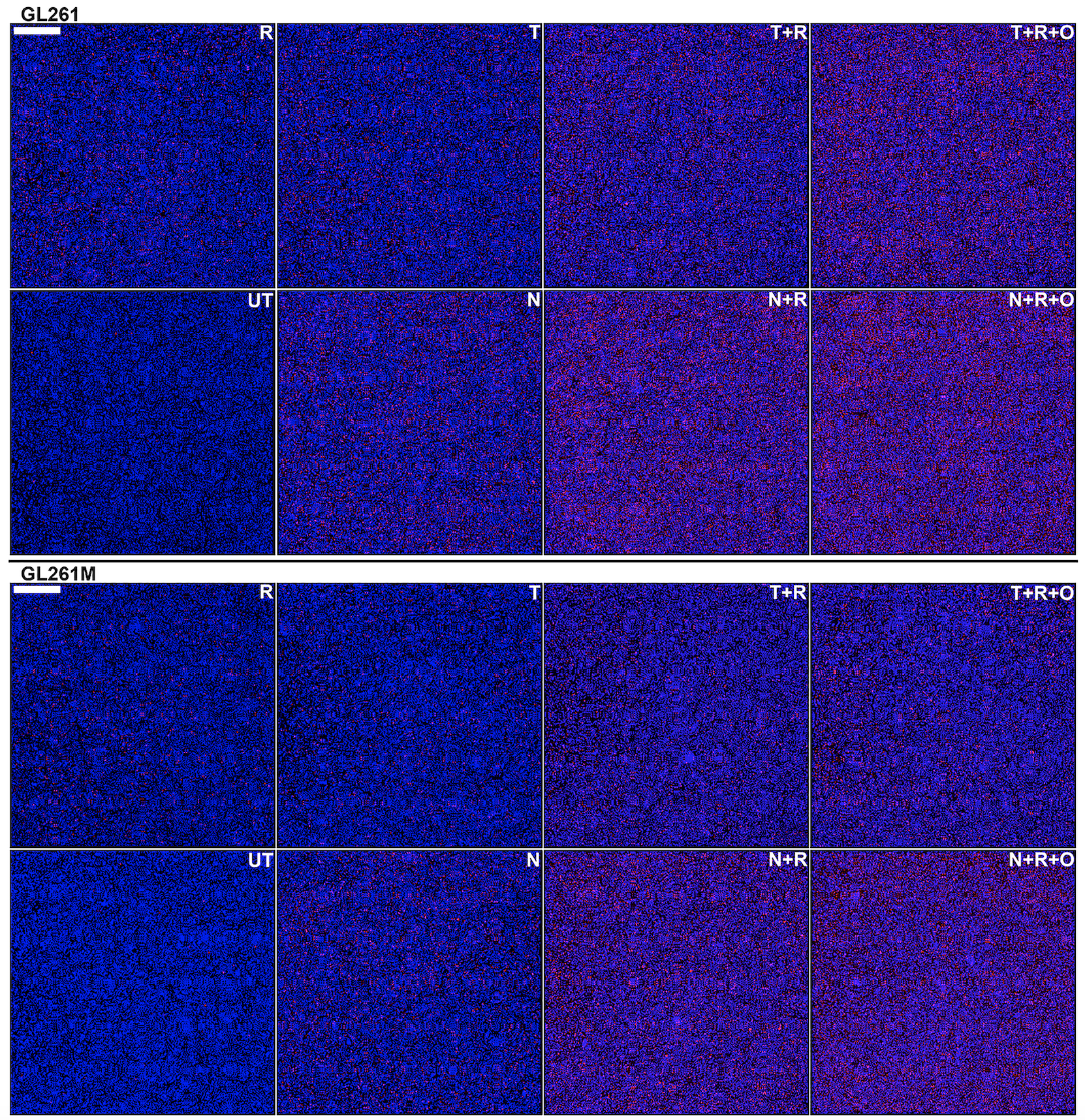

Supplement: S6 Fig — Cells were seeded at high densities (50,000 cells/cm2) and either left untreated (UT) or treated for five consecutive days with either 10 μM TMZ (T) or NEO212 (N) or 2 Gy (R) alone or combinations without (T+R or N+R) or with (T+R+O or N+R+O) Olaparib (O). The cells were probed with a γH2AX antibody and an AF647-labeled secondary and nuclei were counterstained with DAPI. Persistent γH2AX foci (red) were digitally counted relative to the total number of cell nuclei (blue). Each panel is data dense and represents a composite of 36 fields in total (i.e., a square of about 3x3 mm) captured on a widefield microscopy instrument and digitally stitched together. Scale bar is 500 μm. (TIF) [file pone.0238238.s006.tif]

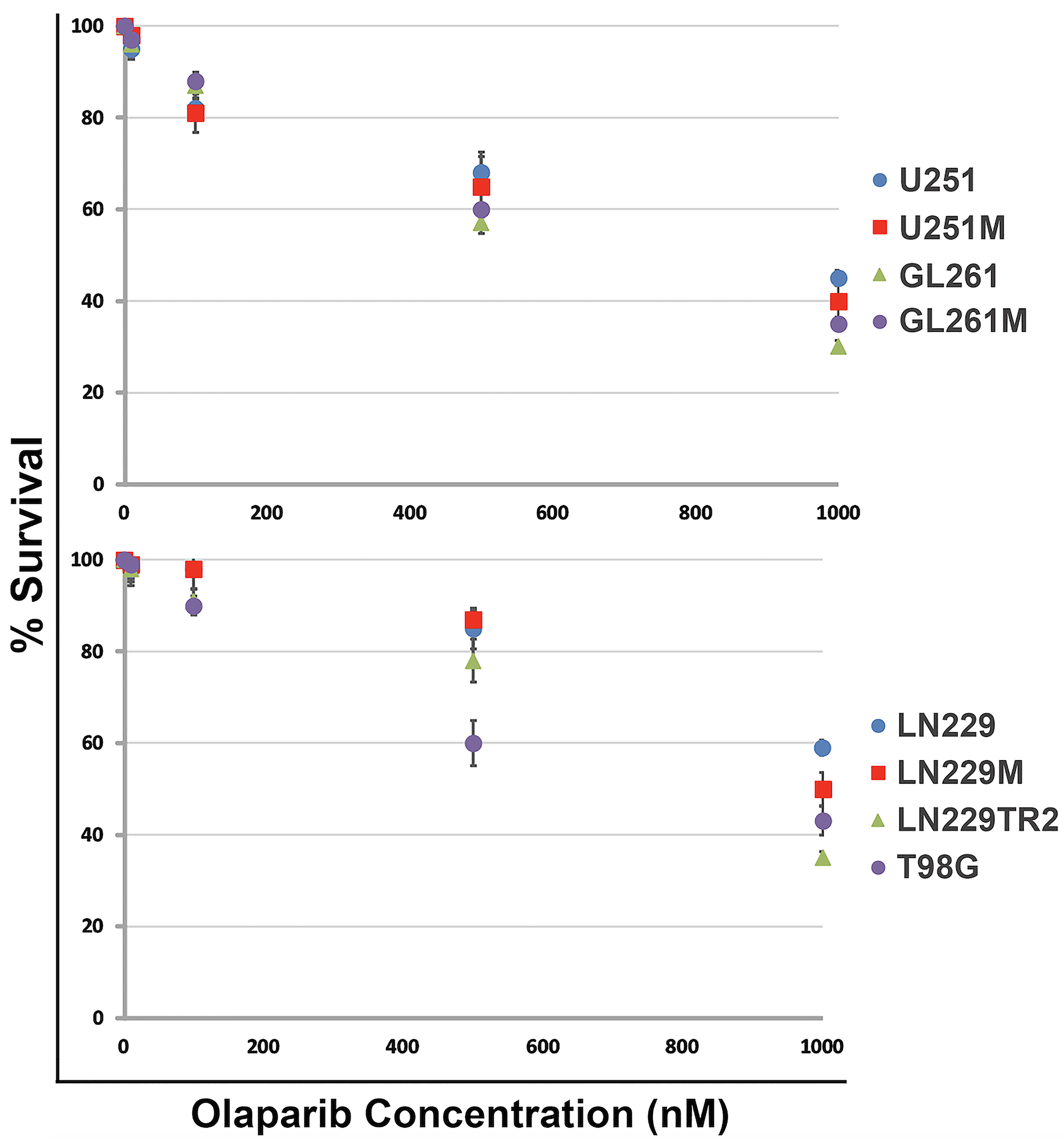

Supplement: S7 Fig — In these CFA analyses, we tested Olaparib on all GB cell lines over a range of concentrations (0–1000 nM). The colony survival data show that Olaparib is barely cytotoxic in the lower end of this concentration range. (TIF) [file pone.0238238.s007.tif]

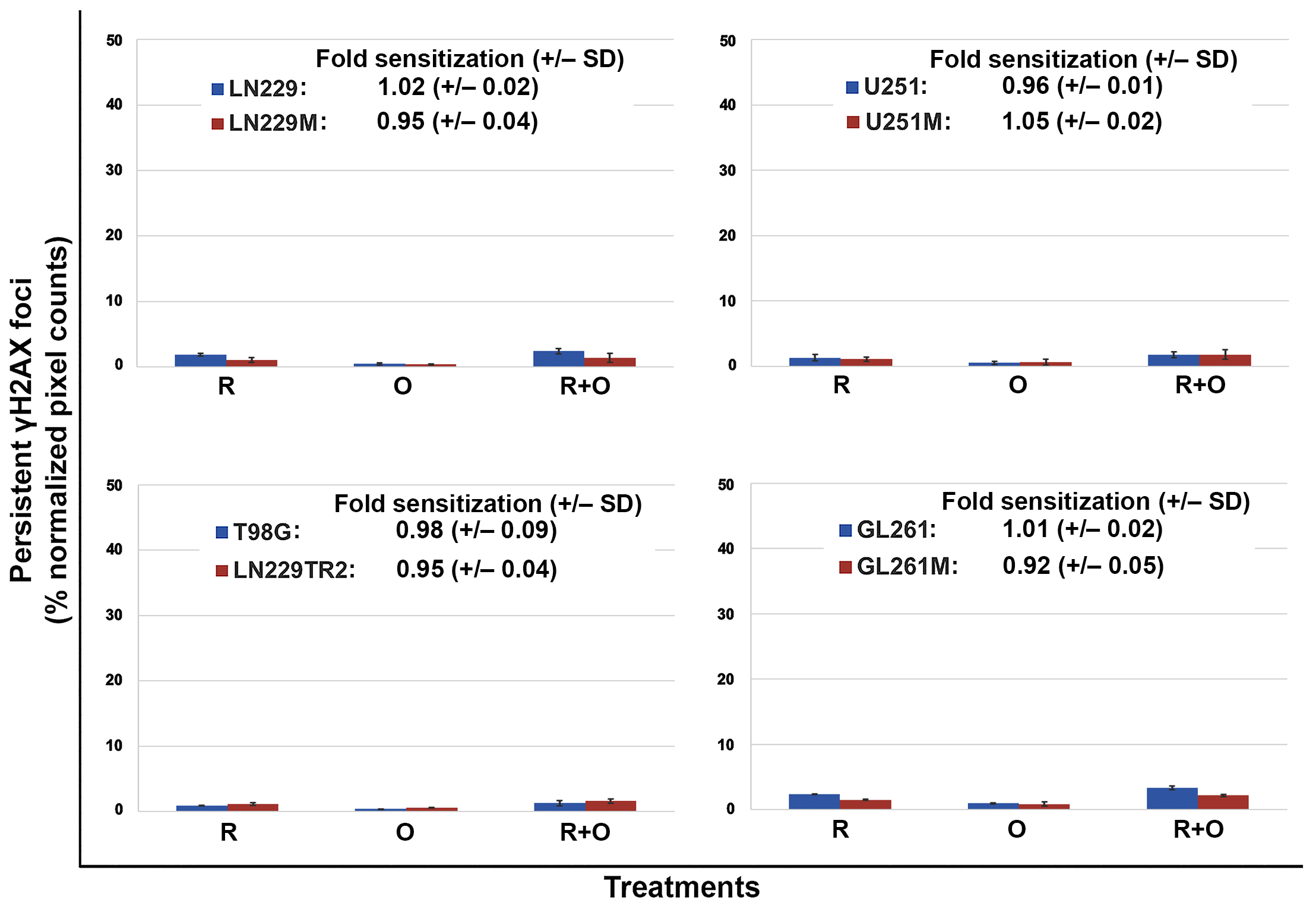

Supplement: S8 Fig — Cells were seeded at high densities (50,000 cells/cm2) and either left untreated or treated for five consecutive days with either 10 nM of Olaparib (O) or 2 Gy (R) alone or the combination of both (R+O). Persistent γH2AX foci were digitally counted and expressed relative to the total number of cell nuclei. All ratio values (pixel counts from treatments expressed as a % ratio of persistent γH2AX foci to nuclei values normalized to untreated counts) were found statistically significant with p values of <0.01 (ANOVA with Tukey post-hoc testing). A fold sensitization value of 1 or close to 1 signifies additive, non-synergistic effects. We deliberately kept the scale of the y-axis the same size for all γH2AX quantifications throughout the study for comparison purposes. (TIF) [file pone.0238238.s008.tif]

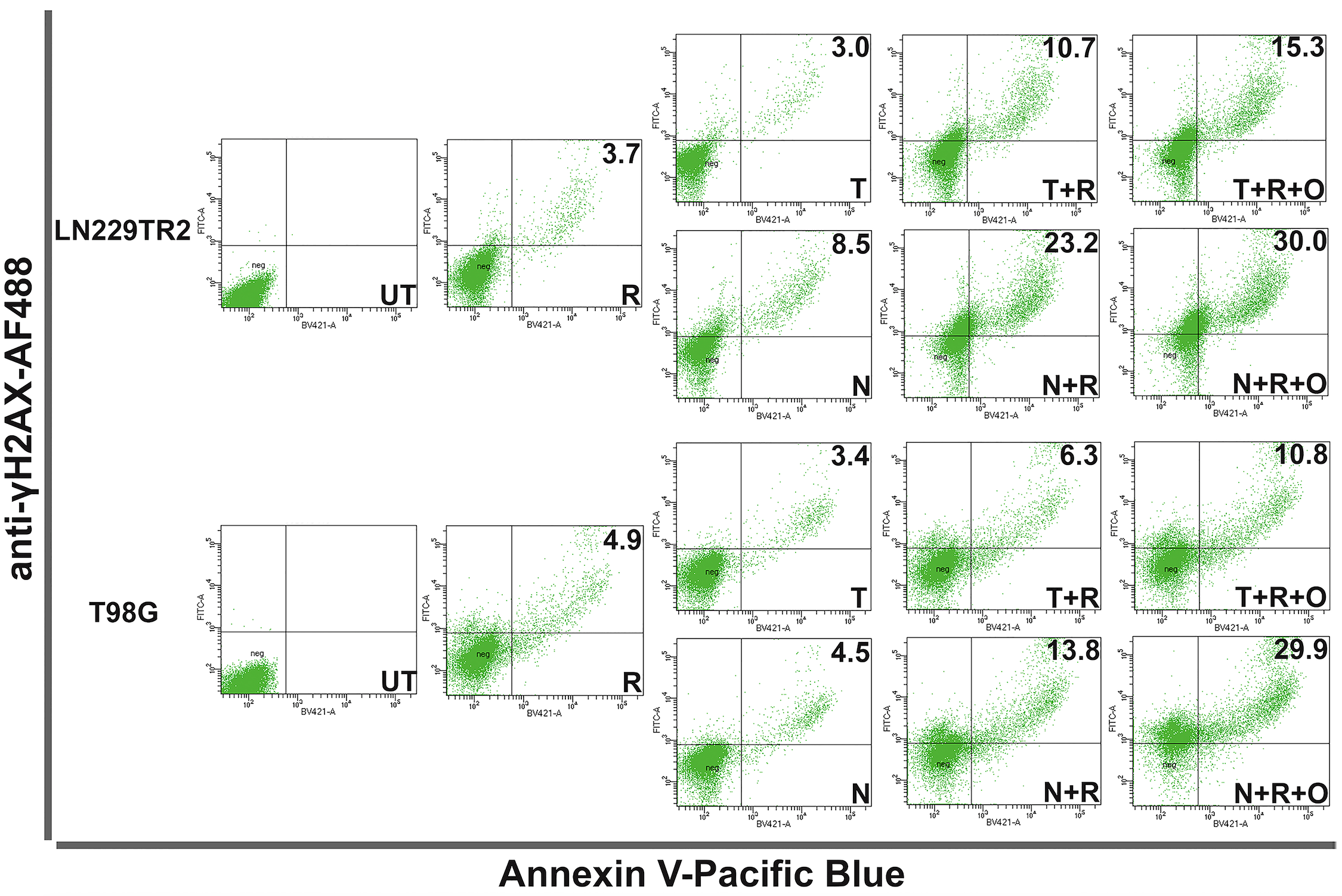

Supplement: S9 Fig — LN229TR2 and T98G cells were seeded at high densities (50,000 cells/cm2) and were either left untreated (UT) or treated for five consecutive days with either 10 μM TMZ (T) or NEO212 (N) or 2 Gy (R) alone or combinations without (T+R or N+R) or with (T+R+O or N+R+O) Olaparib (O). The cells were probed with a Pacific Blue-labeled Annexin V and then fixed, permeabilized and probed with an AF488-labeled γH2AX antibody. γH2AX/Annexin V double positive cells (i.e., dead cells due to irreparable DNA damage) are shown as percentages of total cell numbers. Representative panels are shown from three independent experiments. (TIF) [file pone.0238238.s009.tif]

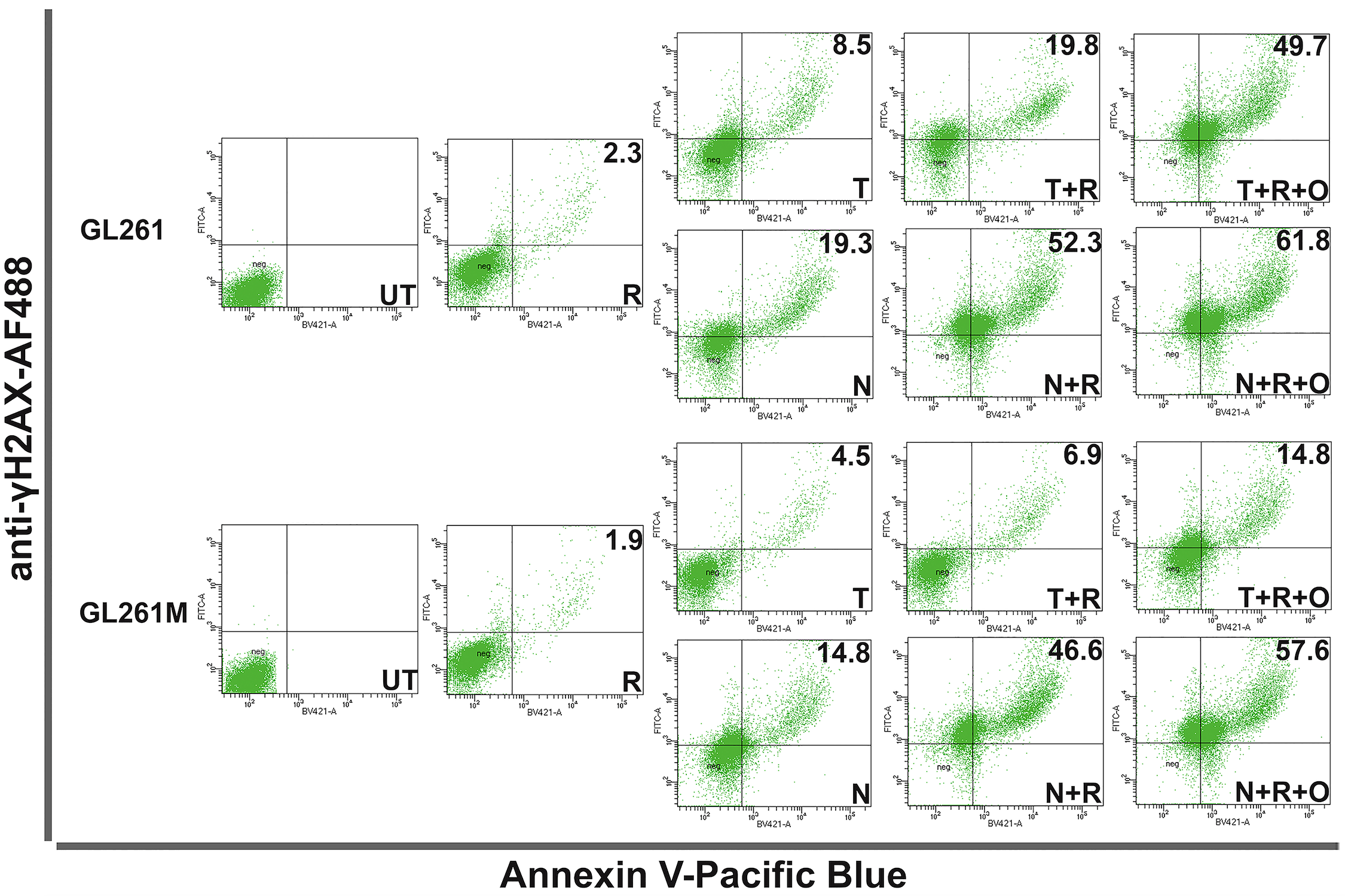

Supplement: S10 Fig — GL261 and GL261M cells were seeded at high densities (50,000 cells/cm2) and were either left untreated (UT) or treated for five consecutive days with either 10 μM TMZ (T) or NEO212 (N) or 2 Gy (R) alone or combinations without (T+R or N+R) or with (T+R+O or N+R+O) Olaparib (O). The cells were probed with a Pacific Blue-labeled Annexin V and then fixed, permeabilized and probed with an AF488-labeled γH2AX antibody. γH2AX/Annexin V double positive cells (i.e., dead cells due to irreparable DNA damage) are shown as percentages of total cell numbers. Representative panels are shown from three independent experiments.] (TIF) [file pone.0238238.s010.tif]

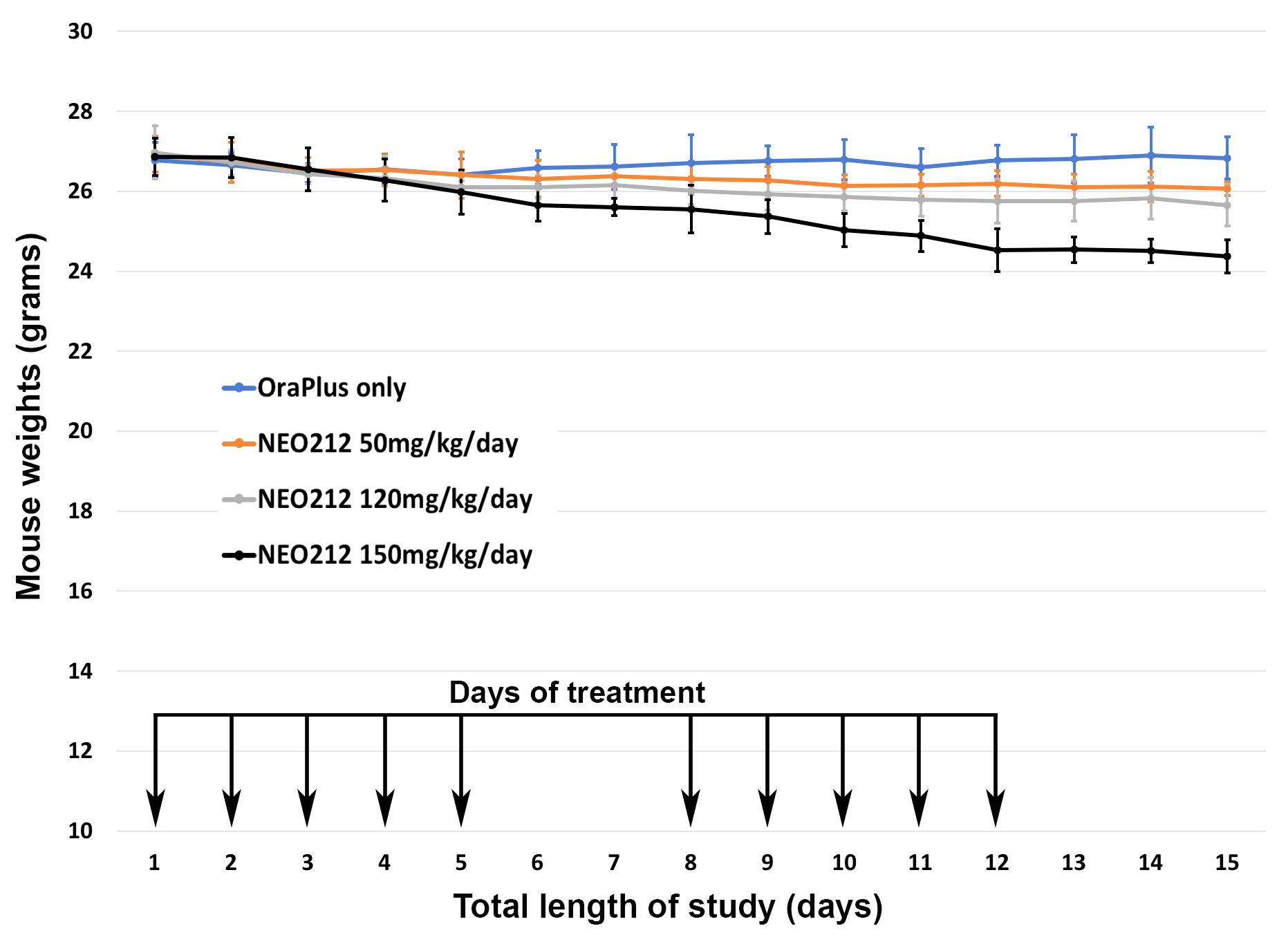

Supplement: S11 Fig — NEO212 appears to be well tolerated by the animals with minor weight loss observed at clinically relevant doses (i.e., 50 mg/kg/day) given orally over 2 weeks (using a schedule of administration of 5 days on/2 days off). At higher dosages (i.e., 150 mg/kg/day) of NEO212, the change in weight appears to be more pronounced, with a loss of about 10% of the total body weight at the beginning of the study. (TIF) [file pone.0238238.s011.tif]

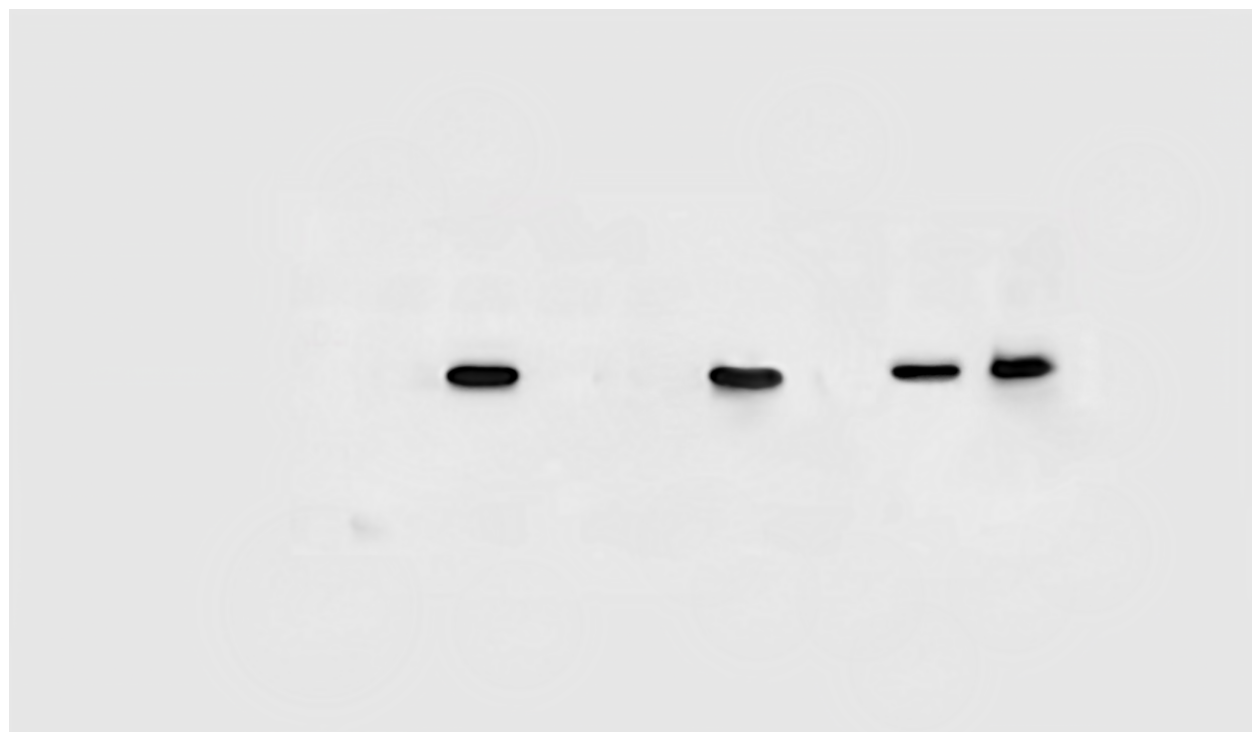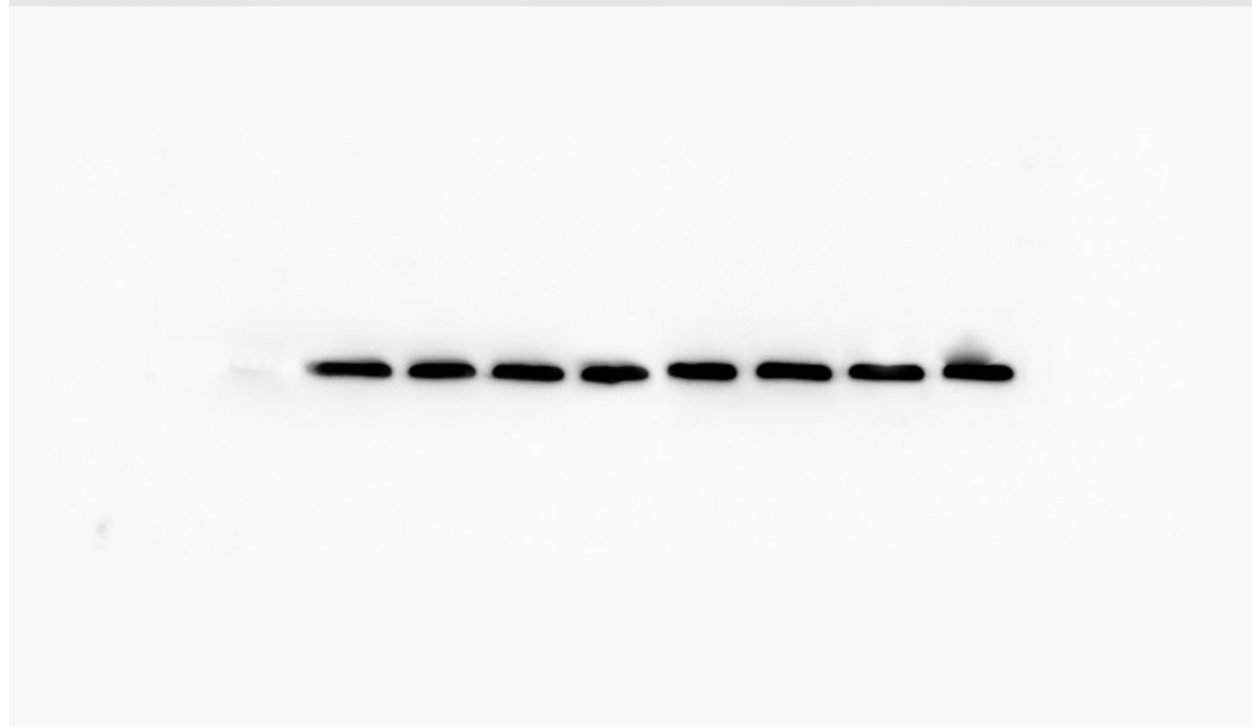

Supplement: S1 File — (PDF) [file pone.0238238.s012.pdf]
