# Supplementary material for: Developing a clinically relevant radiosensitizer for temozolomide-resistant gliomas
Source: PLoS One. 2020 Sep 3;15(9):e0238238. doi: 10.1371/journal.pone.0238238 (PMC7470340; doi:10.1371/journal.pone.0238238)
Supplement: Attachment — Submitted filename: Response to Reviewers.pdf [file pone.0238238.s013.pdf]

USC
UNIVERSITY
OF SOUTHERN
CALIFORNIA

Keck School of Medicine

Department of Neurological Surgery

Radu O. Minea, M.D.
Research Assistant Professor

University of Southern California
Los Angeles, California 90089
Tel: 323-442-5680
e-mail: minea@usc.edu

July 16, 2020

Robert W. Sobol, PhD
Academic Editor, PLOS ONE

Dear Dr. Sobol,

I am addressing this letter to you as the Academic Editor and to the reviewers of our manuscript (PONE-D-20-13771) entitled *'Developing a clinically relevant radiosensitizer for temozolomide-resistant gliomas'*. We would like to thank you all for the time you took to review our manuscript and for your insightful critiques and helpful comments.

As requested by the Journal, we have now added the uncropped gel/blot images for the Western blot analyses (in Figure 3) to the Supporting Information section of the Journal. Unfortunately, the original images of our Western blot analyses on the MGMT expression levels of our glioblastoma (GB) cell line models were cropped at the time of their original capture by our graduate student on the imager. For this reason, we decided to repeat these WB analyses and we are now in the position to provide uncropped gel/blot images for these as well as a rebuilt Figure 3 in the revised manuscript.

Please also note that we have also addressed your concerns regarding the "data not shared" phrase used in the original submission of the manuscript. The phrase was used in relation to our radiosensitization data generated with minimally cytotoxic concentrations of Olaparib, a PARP inhibitor. To alleviate this concern, we decided to assemble these data in the form of a new supplemental figure (i.e., Fig. S8), now included in the revised manuscript. Please do note that because of this, the numbering of the figures following the new Fig. S8 has changed accordingly (e.g., previous Fig. S8 is now Fig. S9 and so on).

Lastly, for the sake of clarity, we have further streamlined the Abstract down to 300 words and operated additional edits throughout the manuscript in an effort to further improve the reader's experience. As requested, all these can now be found in a separate marked-up pdf copy of our manuscript that highlights all the changes made to the original version.

As far as the specific comments made by the reviewers (*listed in italics*), these are addressed point by point below:

Reviewer #1:
"Although there is interesting and promising data shown, there is a concern with the major premise that could be addressed more rigorously. The premise that NEO212 has enhanced activity because it is producing more DNA adducts, specifically N-methylpurine adducts, which then overwhelms BER and can also radiosensitize. It is a reasonable assumption, but it is inferential because methyl purine adducts have not been measured in this or other previous studies."

<u>Response</u>: Although we fully agree with all the points raised by the reviewer, measuring the methylpurine adducts generated by NEO212 is not trivial. One sensitive method for measuring all adducts (rather than just the N-methylpurine ones) is to use radiolabeled

versions of both NEO212 and TMZ. This approach allows for head-to-head comparisons between the methylation potential of both molecules at equivalent dosages. On the other hand, the level of specific N-methylpurine adducts can be estimated by measuring the BER intermediates generated by these molecules through methods described by Strom et al. in a paper cited by us (reference #50 in our manuscript). We are currently looking into ways to run all these experiments with NEO212. In the meantime, we have just been awarded an STTR Phase I grant by the NCI (with Dr. Chen as the PI of the grant and Dr. Minea as the PI of the USC subcontract) in which we proposed among other things to generate ^{14}C-labeled versions for both TMZ and NEO212. Our plans are to label both molecules with the ^{14}C radioisotope, but only at the methyl group that is donated by these two alkylating agents to DNA (via the methyldiazonium ion). This important work has just started, and we are confident that we will be able to get a better understanding of the differential in DNA methylation between the two drugs at clinically relevant concentrations (i.e., 10 µM or less). This is expected to generate a far more precise readout of the (i) tumor cell uptake, and (ii) DNA methyl damage achieved comparatively with NEO212 vs. TMZ in vitro and in various organs in animal models. Along these lines, all quantifications in these biodistribution/tissue methylation studies conducted in vitro and in vivo with ^{14}C-NEO212 and ^{14}C-TMZ will be all done in a liquid scintillation counter.

Keck School of Medicine

Department of Neurological Surgery

Radu O. Minea, M.D.
Research Assistant Professor

Reviewer #1:
"Related, a key sentence stating the adduct distribution for TMZ and NEO212 is the same, in lines 313-315 goes unreferenced. Moreover, the reference to synthesis and characterization of NEO212 elsewhere in the manuscript, #28, is actually a review with some in silico analysis but no apparent in vitro or cellular analysis of DNA base adduct distribution. The authors should provide the data or clearly cite a reference, because this is at the core of argument for the mechanism of action for NEO212, its enhanced penetration into cells and across the BBB. This data would enhance the rigor supporting the hypothesis."

Response: We agree that our previous statement on the adduct distribution pattern for TMZ and NEO212 is currently not supported by direct experimental data and it can only be inferred from our indirect experimental data generated comparatively with both molecules. For this reason, we have amended the statements in question (i.e., the original 313-315 lines) in the revised manuscript to better reflect the current evidence we have with NEO212. It is worth noting however that we have provided convincing evidence based on our GC/MS analyses in support of the notion that (i) NEO212 breaks intracellularly to release intact TMZ and (ii) the amount of TMZ released by NEO212 inside the cells is higher than the amount of TMZ taken up by the cells when cells are incubated with either drug at the same dosages. Moreover, our experiments with decayed NEO212 (for which we added further clarifications in the revised manuscript) show that NEO212 'decays' or breaks down in aqueous solutions in a very similar fashion to what has been previously reported with TMZ. These indirect lines of evidence seem to argue that the cytotoxic mechanism by which both molecules operate is identical and it exclusively resides with their ability to release the highly reactive methanediazonium chemical species which further alkylates DNA. Lastly, as stated above, we are currently funded by the NCI to generate radiolabeled species for both molecules which we hope will provide a more direct proof that NEO212 can generate significantly more DNA adducts than TMZ at equivalent dosages.

University of Southern California
Los Angeles, California 90089
Tel: 323-442-5680
e-mail: minea@usc.edu

Reviewer #1:
"The experiments with "decayed" NEO212 in supplementary Figures S3 and S4 are insufficiently explained."

USC
UNIVERSITY
OF SOUTHERN
CALIFORNIA

Keck School of Medicine

Department of Neurological Surgery

Radu O. Minea, M.D.
Research Assistant Professor

Response: We have further edited that section of the manuscript to better explain the experiments conducted with decayed NEO212. We hope that this was addressed to the satisfaction of the reviewer.

Reviewer #1:
"The data presented in the manuscript supporting DNA damage, immunofluorescence of gamma-H2AX after a 24 hour time point is inferential."

Response: We agree that the ICC γH2AX data is inferential (i.e., more correlative than mechanistic in nature). However, the significance of γH2AX foci is very well understood and accepted by the scientific community and was appropriately referenced by us. The kinetics of γH2AX foci are also published. The significance of the persistence of the γH2AX signal at 24 hours after the application of the genotoxic insult (i.e., irreparable DNA damage) has also been acknowledged by the people in the field and was also appropriately referenced by us. We strongly believe that, as a whole, our quantitative ICC data generated with NEO212 and TMZ provides plenty of evidence in support of our assertions that NEO212 is more potently cytotoxic and has a superior radiosensitization profile compared to TMZ. The methods used by us in our study are also widely employed by many research groups when studying the extent of DNA damage inflicted by alkylating agents such as TMZ. In all these studies, the extent of DNA damage is expressed indirectly as a γH2AX readout.

Reviewer #1:
"The images in Figures 5 and 6 are poor quality, may just be the PDF. The pixilation does not provide confidence in the quantitation shown in Figure 7. The Figure legend, lines 451 and 459, state scale bar is 200 microns, but there are no scale bars on the images."

Response: These are related points and we are addressing them together. We strongly encourage the reviewers to follow the links provided by the PLOS ONE journal and download the high-resolution versions of **all** of our figures (especially the qICC ones). These images are not very large in size (all are under 10 MB) and are readily accessible even with slower internet connections. We totally agree with the reviewer that the figures assembled (automatically by the Journal) in the pdf file of the manuscript are extremely low resolution and, because of this, no reasonable assessment can be drawn on the significance of these data from these images. Furthermore, to help with their visualization, we have modified the scale bars in our qICC figures by making them larger and thicker. The scale bars are now set at 500 microns and are easier to see. Lastly, please note that each panel (i.e., each treatment condition) from our multi-panel qICC figures is data dense and captures the composite information from an area with the size of about 3x3 mm, which is essentially a third of a well from a 96-well plate. This is a huge amount of information which makes our ICC strategy a highly quantitative one.

Reviewer #1:
"There is a line in the figure legend of Figure 3 that does not make sense, nor does the corresponding data in Figure 3 with O6BG. Line 374-76. "Inclusion of O6BG improved the survival." Benzylguanine covalently inactivates the MGMT protein. This inactivation should SENSITIZE cells because there are hundreds of papers that show that MGMT protein activity is protective against the methyl damage caused by TMZ and mechanistically similar methylating agents. Or O6BG has no effect in cells lacking MGMT."

University of Southern California
Los Angeles, California 90089
Tel: 323-442-5680
e-mail: minea@usc.edu

USC
UNIVERSITY
OF SOUTHERN
CALIFORNIA

Response: This mistake was unintended, and we apologize for missing it. We have now amended our original statement regarding O6BG to reflect the true effect of this inhibitor (i.e., the sensitization of MGMT-positive tumor cells to DNA methylating agents).

Reviewer #1:
"Line 333, Figure 2 does not have a figure legend."

Response: We are not sure what happened here. We checked the online version of the manuscript (on the PLOS ONE website) and the legend for Figure 2 was indeed there. Furthermore, we have double-checked the revised version to make sure that all figure legends were included in the text.

Reviewer #1:
"Line 98, MGMT is a suicide protein, not an enzyme."

Keck School of Medicine

Department of Neurological Surgery

Radu O. Minea, M.D.
Research Assistant Professor

Response: We agree with this statement and we made all the appropriate corrections in the manuscript regarding the function (i.e., not a true enzyme) of this DNA repair protein.

Reviewer #1:
"Line 105. "potentially" or "potently"? The cytotoxicity of O6-methylguanine is well established."

Response: We agree and removed the word "potentially" altogether to clear this ambiguity.

Reviewer #2:
"Because the study involved in mouse model, a tumor growth curve, mouse survival curve or endpoint tumor size images comparing TMZ or NEO211 treatment is needed to support the conclusion better. The mouse body weight curve is needed to indicate the systemic toxicity."

Response: We totally agree with the reviewer's statement regarding the needed animal tumor efficacy and survival studies. As explained above, however, we are currently funded by the NCI to conduct systematic in vivo biodistribution, pharmacokinetics, neurotoxicity, and tumor efficacy studies with NEO212 in animal models of GB. These studies have just begun, and we anticipate, in light of the current COVID-19 situation that these will advance at much slower pace than we had originally anticipated or hoped for. It may take many more months before we will be able to generate some of these data. Because of this, we respectfully ask the reviewers to allow the publication of our manuscript in its current form, for which we had already generated a significant amount of data. To address the reviewer's point regarding the mouse body weight curves during our preliminary toxicity studies, we have now added this info in the form of a supplementary figure (i.e., Fig. S11) in the revised manuscript.

Thank you for the comments,

Radu Minea

Radu O. Minea, M.D.

University of Southern California
Los Angeles, California 90089
Tel: 323-442-5680
e-mail: minea@usc.edu
